# Functional outcomes of three-corner fusion without triquetrum excision versus conventional four-corner fusion in scaphoid non-union advanced collapse G II and III in active patients: a prospective randomized control trial

**DOI:** 10.1051/sicotj/2024052

**Published:** 2024-12-12

**Authors:** Khaled Nabil Youssef, Amr Nabil, Ahmed Naeem Atiya, Mohammed Mostafa El-Mahy

**Affiliations:** Orthopedic Department, Faculty of Medicine Ain Shams University 38 Abbassia Square Cairo 1181 Egypt

**Keywords:** Scaphoid fracture, Four-corner fusion, SNAC wrist, Three corner fusion

## Abstract

*Introduction*: Four-corner fusion has long been the preferred treatment for stages II and III of scaphoid nonunion advanced collapse with intact radiolunate articulation. Three corner fusions were then proposed as a more limited procedure with improved ulnar deviation through triquetrum excision. However, we believe triquetrum preservation would decrease the radiolunate contact pressure without affecting the ulnar deviation range. *Methods*: This prospective randomized study was performed between March 2019 and May 2021 and involved a total of 34 patients who underwent four corner fusions or three corner fusions without triquetrum excision for SNAC grade II and III. The average follow-up period was 2 years. Follow-up included radiological and clinical evaluation (range of motion, grip strength, visual analogue scale, and modified Mayo wrist scores). *Results*: There were no significant differences between the two groups as regards the postoperative range of motion, grip strength, visual analogue scale, modified Mayo wrist scorers, and complication rate. However, the three-corner fusion group had less mean operative time compared to the four-corner fusion (mean ± SD; 77.6 ± 16.9, 103.8 ± 10.2 min – *P* < 0.001) respectively. *Conclusion*: The authors concluded that three-corner fusion without triquetrum excision offered a comparable functional outcome and complication rate to four-corner fusion with less operative time in the three-corner fusion group.

## Introduction

Scaphoid bone fractures are considered one of the most common injuries to the upper limb, with an estimated incidence of 1.47 fractures per 100,000 person-years in the United States and accounts for 60% of carpal bone fractures [1, 2]. In acute cases, approximately 40% of cases are missed at initial presentation [3]. Therefore, a certain number of acute fractures are not diagnosed during the injury and remain undiagnosed until symptoms of non-union appear. The estimated non-union rates may reach up to 15% [1]. Longstanding scaphoid nonunion results in carpal bone instability and progressive collapse followed by radiocarpal osteoarthritis development, known as scaphoid nonunion advanced collapse (SNAC) [4].

In the SNAC wrist, wrist degeneration starts at the radial styloid-scaphoid articulation and then progresses to the entire radio-scaphoid joint, ultimately involving the lune-capitate joint [5]. Several motion-salvage procedures have been described for SNAC wrist depending on its stage. Consistent radio-lunate joint sparing is the main prerequisite for such procedures. The most used surgical procedures for advanced stages include scaphoid resection with midcarpal arthrodesis or proximal row carpectomy [6].

The first reported midcarpal arthrodesis was done by Thornton in 1924 for a case of neglected capitate dislocation. He excised the base of the capitate and underwent fusion between the scaphoid, lunate, capitate, and hamate [7]. The unique concept of four-corner fusion was first described by Watson in 1984, which includes a scaphoidectomy and a fusion between the capitate, lunate, hamate, and triquetrum [5]. A more limited three-corner arthrodesis was described, in which, the capitate, lunate and hamate were fused and the excised scaphoid and radial styloid were the main sources of graft. An improvement in grip strength from 59% to 64% was reported [7]. Delattre et al., suggested additional triquetrum excision to improve ulnar deviation, and this improvement was reported to be better than triquetrum preservation as in 4CF in another clinical study [6, 8]. Other motion salvage procedures include scaphoidectomy and lunocapitate fusion, bicolumnar fusion or midcarpal tenodesis [9–11].

Several studies have reported the value of triquetrum excision during limited wrist fusion, including two cadaveric studies which reported improved ulnar deviation [12, 13]. However, the clinical studies reported less improvement in ulnar deviation than cadaveric studies [6, 8, 14]. To our knowledge, no prospective studies are comparing the outcomes of triquetrum preservation with triquetrum excision or with 4CF.

Our study aims to compare three-corner fusion without triquetrum excision versus four-corner fusion as regards operative time, functional outcomes, and complication rates.

## Materials and methods

We recruited 34 active patients aged between 18 and 55 years old with SNAC grades II and III. Patients were randomized using a computer-generated sequence into two groups, one group had three corner fusions without triquetrum excision (3CFT) (group A) and the other group had conventional four corner fusions (4CF) (group B). We excluded patients with pancarpal arthritis and other carpal bone pathologies e.g. Keinbock disease. The primary outcomes were wrist range of motion, grip strength and wrist functional score, while the secondary outcome was the operative time. A detailed history and physical examination of both wrists were obtained, especially emphasizing the range of motion and grip strength using a dynamometer compared to the normal side with assessment according to the Mayo modified wrist score (MMWS) [10]. The functional outcome was ranked as excellent (90–100), good (80–89), fair or satisfactory (60–79), or poor (<60). The dynamometer used for handgrip strength was the Baseline^®^ Pneumatic Squeeze Bulb Dynamometer (manufactured by Fabrication Enterprises, Inc., NY, USA) which measures the power grip by pounds per square inch (PSI). The average of three successive measures was taken with the arm adducted, elbow flexed 90° and hand in a neutral position to measure the power grip. Preoperative radiographs were evaluated for the SNAC grade and the presence of any extension of the lunate (DISI).

**Figure 1:**
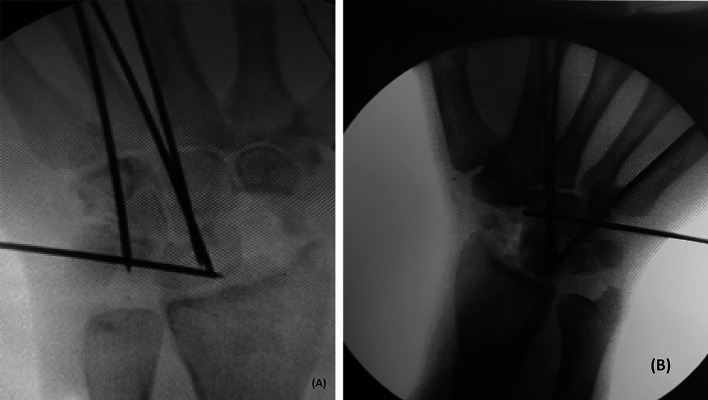
Intraoperative image after fixation by K-wires. (a) Four corner fusion; (b) Three corner fusion without triquetrum excision.

All patients operated through the standard dorsal ligament-sparing approach after the identification of certain palpable landmarks as described by Berger et al. [15, 16]. After the assessment of the radiolunate articular cartilage, the non-united scaphoid was excised, and then the radioscaphocapitate ligament was assessed for its integrity. According to the planned procedure, the articular cartilage between carpal bones was denuded and carpal bones fused using K-wires after reduction of the capitolunate axis if needed (Figures 1a and 1b). Autologous bone graft was obtained either from resected scaphoid, distal radius, or iliac crest as needed. Before closing the capsule, an intraoperative assessment of a passive range of motion in both sagittal and coronal planes was done. Radial styloidectomy, dorsal lunate osteophyte or dorsal radius ridge resection were performed in cases of limited radial deviation or extension secondary to impingement, respectively. A forearm removable splint was applied for 2 weeks, then removable orthosis was applied for 6–8 weeks. Once bone consolidation was achieved, active and passive exercises were allowed. The K-wires were removed 2–3 months postoperatively. Then patients were followed up every 3 months to assess the development of any complications for one year and then every 6 months for another year.

## Results

We included a total of 34 patients, 17 in each group. The mean age was 37 years and 43.06 years in the 4CF and 3CFT groups, respectively. All cases were males, this could be explained by that most manual workers and drivers in Egypt are males, and these occupations more than 70% of the study population. Otherwise, we had no significant differences in baseline demographics data except for age, but without clinical significance as shown in Table 1. The preoperative measurements were homogenous between the two groups regarding power grip, MMW score, range of motion and VAS score with no statistically significant difference between the two groups.

**Table 1:** Demographic data.

	Four corner (4CF) (*N* = 17)		Three corner (3CFT) (*N* = 17)		t*	*P*-value
	Mean	SD	Mean	SD		
Age	37.00	7.91	43.06	8.63	2.13	0.04 S

	*N*	%	*N*	%	χ^2**^	*P-*value

Occupation						
Manual worker	9	52.9%	10	58.8%	1.79 FE	0.43 NS
Driver	5	29.4%	2	11.8%		
Other	3	17.6%	5	29.4%		

* Student’s *t*-test.

** Chi-square test.

4CF: Four corner fusion, 3CFT: three corner fusion without triquetrum excision, S: significant, NS: Not significant.

However, we found that the mean operative time in the 3CFT group was 77.65 min in comparison to 103.82 min in 4CF and it was statistically significant and only one case needed graft from outside the carpal bones (Table 2). Intraoperative assessment of passive ulnar deviation showed no statistically significant difference between 3CFT and 4CF with mean range 35°–32° respectively.

**Table 2:** Operative data.

	Four corner (4CF) (*N* = 17)		Three corner (3CFT) (*N* = 17)		t*	*P*-value
	Mean	SD	Mean	SD		
Operative time (min)	103.82	10.24	77.65	16.87	5.47	<0.001 HS

	*N*	%	*N*	%	χ^2**^	*P*-value
Graft outside carpal						
Yes	10	58.8%	1	5.9%	10.89	0.001 HS
No	7	41.2%	16	94.1%		

* Student’s *t*-test.

** Chi-square test.

4CF: Four corner fusion, 3CFT: three corner fusion without triquetrum excision, min: minutes, HS: High significant.

**Figure 2:**
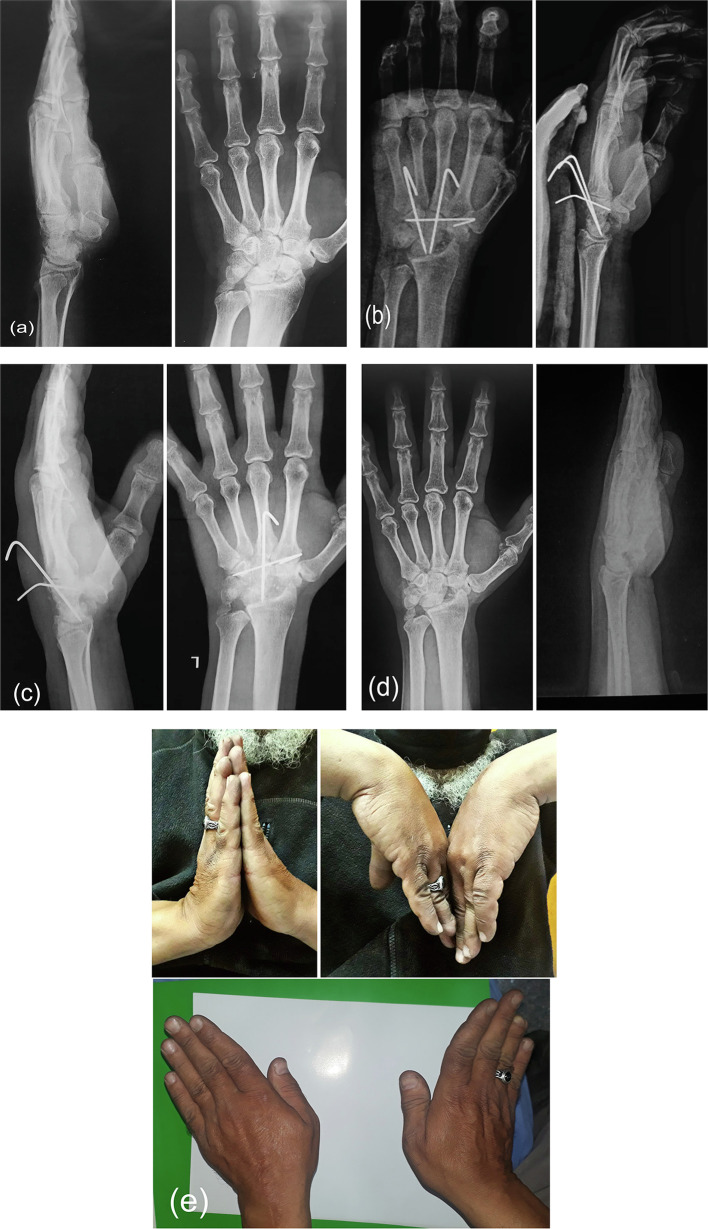
Case from 3CFT. (a) Preoperative X rays, show SNAC stage III; (b) Immediate postoperative X-rays, with correction of DISI deformity on lateral radiographs; (c) At 8 weeks follow-up, with good union between carpal bones; (d) On of the wires was removed as it became loose; (e) After one-half year follow-up.

At 2 years follow-up, both surgical procedures resulted in significant improvement in all functional outcomes and VAS scores. However, the range of motion decreased in both study groups which was expected following limited wrist arthrodesis (Figure 2). The 3CFT group had a more statistically significant decrease in radial deviation than the 4CF group, but it was of no clinical significance (Table 3).

**Table 3:** Preoperative and postoperative assessment of both groups on follow-up.

	Preoperative						Postoperative					
	Four corner (4CF) (*N* = 17)		Three corner (3CFT) (*N* = 17)		t*	*P*-value	Four corner (4CF) (*N* = 17)		Three corner (3CFT) (*N* = 17)		t*	*P*-value
	Mean	SD	Mean	SD			Mean	SD	Mean	SD		
Power grip (PSI)	8.24	1.60	7.88	1.41	0.68	0.50 NS	10.65	1.84	10.24	1.60	0.70	0.49 NS
Percent	0.63	0.07	0.65	0.05	1.09	0.28 NS	0.82	0.05	0.85	0.08	1.09	0.28 NS
Flexion	45.00	8.10	44.41	9.17	0.20	0.84 NS	37.06	8.11	36.76	8.28	0.11	0.92 NS
Extension	41.47	8.06	44.12	8.34	0.94	0.35 NS	33.53	8.62	35.59	9.50	0.66	0.51 NS
Radial deviation	8.53	3.43	9.59	4.15	0.81	0.42 NS	8.24	3.03	6.76	3.03	1.41	0.17 NS
Ulnar deviation	28.53	7.24	27.06	8.30	0.55	0.59 NS	24.71	5.72	20.88	6.67	1.79	0.08 NS
VAS	6.47	.62	6.35	0.79	0.48	0.63 NS	2.65	0.70	2.88	0.86	0.88	0.39 NS
MMW	49.12	4.04	47.65	7.73	0.70	0.49 NS	68.24	5.29	70.59	8.64	0.96	0.35 NS

* Student’s *t*-test.

4CF: Four corner fusion, 3CFT: three corner fusion without triquetrum excision, S: significant, NS: Not significant, PSI: Pounds per square inch, VAS: Visual analogue scale, MMW: Mayo modified wrist score.

Two cases, one in each group, developed lunocapitate nonunion and early radiolunate arthritis. The case from the 3CFT group underwent total wrist arthrodesis, however the case from 4CF refused to undergo the further procedure.

## Discussion

Scaphoid nonunion advanced collapse is a common first presentation for scaphoid fracture, as more than one-third of acute scaphoid fractures are missed on initial radiographs [3]. Several motion salvage surgeries exist for SNAC, in this study we compared the outcomes of 4CF and 3CFT and found statistically significant longer mean operative time in the 4CF group. After an average 2-year follow-up, all functional parameters were significantly improved in both groups. The range of motion was significantly reduced, especially in the sagittal plane; however, it remained higher than the functional range described by Palmer et al. who conducted a biomechanical study using a triaxial electro-goniometer to assess the functional range of motion and reported that 5° flexion, 30° extension, 10° radial deviation, and 15° ulnar deviation, were required for activities of daily living [17].

More than half of the 4CF group needed an additional graft rather than a scaphoid in contrast to the 3CFT group, in which only one case needed additional grafting. No statistically significant difference was found in the time to achieve union between both groups. Approximately 32 cases developed complete union. Thus, usage of the excised scaphoid if good and sufficient bone quality as the source for graft should not be avoided. If the scaphoid was of poor quality, opaque, or not bleeding, another source of bone graft should be used instead. The results of three-corner fusion without triquetrum excision were comparable to the previously published studies reporting different methods of limited wrist fusion (Table 4).

**Table 4:** Postoperative functional parameters of 3CFT, in comparison to previous studies.

Study	Type of study	Type of fusion	Flexion (mean)	Extension (mean)	RD (mean)	UD (mean)	Grip % (contralateral)	VAS	Functional Score	
Vance et al. [1]	Retrospective	4CF Plate = 27, K-wires = 14, staples = 12, screws = 5	30	35	Plate = 5	Plate = 30	Plate = 70	–	Plate = 27 (DASH)	Good to satisfactory
					Others = 10	Others = 25	Others = 80			
Delattre et al. [2]	Retrospective	3CF staples = 10, screws = 14, plate = 6	33	37	13	23	72	–	17 (DASH)	Satisfactory
Gauci et al. [3]	Retrospective	3CF Staples = 27, screws = 1	38	27	8	18	–	2	70 (MWS)	Good
									22 (Quick-DASH)	Satisfactory
Van-Nuffel et al. [19]	Retrospective	4CF 9 = plate	36	29	13	23	–	2.3	19 (DASH)	Satisfactory
Ring et al. [18]	Retrospective, comparative	4CF = 49	Flex/Ext arc 62.4 ± 26.0		RD/UD arc 29.8 ± 10.6		–	3.9 ± 2.6	24.7 ± 10.7 (DASH)	
		3CF/2CF = 9	Flex/Ext arc 62.6 ± 20.5		RD/UD arc 45.0 ± 21.2			2.9 ± 9.8	18.4 ± 9.8 (DASH)	
Current study technique	Prospective, comparative	3CFT K-wires = 17	36	35	6.7	20.8	85%	2.8	MMW = 70	Satisfactory

4CF: Four-corner fusion, 3CF: Three-corner fusion, 3CFT: Three-corner fusion without triquetrum excision, UD: Ulnar deviation, RD: Radial deviation, VAS: Visual Analogue Scale, DASH: The Disabilities of the Arm, Shoulder and Hand, MWS: Mayo Wrist score MMW: Modified Mayo Wrist score.

Two cases reported nonunion between the capitate and lunate of which one K-wire was used between the lunate and capitate and the DISI deformity was not properly corrected. Proper lunate correction to the neutral position has a great value for proper consolidation [20].

The surgical operation for an SNAC wrist mainly aimed to relieve the pain while preserving the maximum range of motion of the radiocarpal joint and maintaining acceptable grip strength and functionality. Over the decades, scaphoidectomy and four-corner arthrodesis were established procedures for SNAC wrist management, with good results in terms of pain relief and grip strength restoration [21]. Some authors have recommended that the capitate be clearly translated to completely cover the distal lunate (lunate-covered position or radial-aligned 4CF) to maximize the surface area of contact between the two bones and prevent proximal migration of capitate on long follow-up [22]. However, biomechanical cadaveric studies compared the anatomic versus radial-aligned 4CF and showed significantly reduced wrist extension and circumduction in radial 4 CF [23].

In cadaveric studies, additional excision of the triquetrum (3CF) provided a greater range of ulnar deviation compared with 4CF. The mean ulnar deviation was 32° and 45°, respectively [12, 13]. This concept was adopted by clinical trials. Two retrospective studies reported a mean ulnar deviation at 23° and 18° following triquetrum excision [6, 14]. Our prospective study, intraoperatively, assessed the ulnar deviation and revealed a mean of approximately 35° which was comparable to the cadaveric studies. On follow-up, the mean was 20° (Table 5). The difference between the cadaveric studies, intraoperative measurements in our study and results of other clinical studies can be explained by the degree of soft tissue contracture and the amount of compensatory hypermobility of the adjacent mobile segments among living subjects. Contrastingly, following the triquetrum excision, the radiolunate contact pressure increased and a proprioceptive function within the wrist could be affected [24, 25].

**Table 5:** Comparison regarding Ulnar deviation range between 3CFT and previously published 3CF with triquetrum excision.

	Type of the study	Reported Ulnar deviation (Mean)
Delattre et al. [6]	Clinical, retrospective	23
Gauci et al. [14]	Clinical, retrospective	18
Bain et al. [12]	Cadaveric	32
Got et al. [13]	Cadaveric	45.87
Current study (3CFT)	Clinical, prospective	Passive, intraoperative = 35
		2-year follow-up = 20.88

We found that K-wires are a satisfactory fixation method and give good results in limited arthrodesis. Several studies discussed different fixation methods in limited wrist fusion. K-wires were found to have comparable outcomes to other fixation methods as regards pain relief, grip strength, range of motion and fusion rate [26]. K-wires give at least a similar union rate with a lower incidence of complex revision surgery and major complications such as impingement compared to the dorsal plate [27, 28]. Hayes et al., conducted a systematic review comparing different fixation methods e.g. K-wires, plates, and stables…in limited wrist fusions and found no difference between these methods, except for the grip strength compared to the contralateral limb, which was lower with a metal locking plate than K-wire [29].

To our knowledge, this is the first prospective clinical trial that compares both techniques. However, our study has certain limitations. The sample size was relatively small, and all the candidates were males. Our findings are encouraging; however, we do not have a long-term comparative follow-up. Although there are no negative sequelae of 3CFT in the short term, we continue to observe those patients carefully.

## Conclusion

Three-corner fusion without triquetrum excision and four-corner fusion had the same good functional results. 3CFT had a shorter operative time without the need to use a further graft rather than the excised scaphoid. Proper correction of the DISI deformity and using at least two K-wires between the lunate and capitate improve the union rate. However, more prospective randomized controlled trials with longer follow-up are needed.

## Data Availability

All data underlying the results are available as part of the article and no additional source data are required.
